# Supplementary material for: Chitosan (CS)/Hydroxyapatite (HA)/Tricalcium Phosphate (β-TCP)-Based Composites as a Potential Material for Pulp Tissue Regeneration
Source: Polymers (Basel). 2023 Jul 28;15(15):3213. doi: 10.3390/polym15153213 (PMC10421191; doi:10.3390/polym15153213)
Supplement: Supplementary file 1 [file polymers-15-03213-s001.zip › polymers-2460107-supplementary.pdf]

polymers

MDPI

Supplementary Materials: Chitosan (CS)/Hydroxyapatite (HA)/Tricalcium Phosphate (β-TCP)-Based Composites as a Potential Material for Pulp Tissue Regeneration

Ingrid Zamora [1], Gilbert Alfonso Morales [1], Jorge Iván Castro [2], Lina Marcela Ruiz Rojas [3], Carlos Humberto Valencia-Llano [1], Jose Herminsul Mina Hernandez [3], Mayra Eliana Valencia Zapata [3] and Carlos David Grande-Tovar [4,*]

[1] Grupo Biomateriales Dentales, Escuela de Odontología, Universidad del Valle, Calle 4B # 36-00, Cali 76001, Colombia; ingrid.zamora@correounivalle.edu.co (I.Z.); gilbert.morales@correounivalle.edu.co (G.A.M.); carlos.humberto.valencia@correounivalle.edu.co (C.H.V.-L.)

[2] Laboratorio SIMERQO, Departamento de Química, Universidad del Valle, Calle 13 No. 100-00, Cali 76001, Colombia; jorge.castro@correounivalle.edu.co

[3] Grupo de Materiales Compuestos, Escuela de Ingeniería de Materiales, Facultad de Ingeniería, Universidad del Valle, Calle 13 No. 100-00, Cali 760032, Colombia; ruiz.lina@correounivalle.edu.co (L.M.R.R.); jose.mina@correounivalle.edu.co (J.H.M.H.); valencia.mayra@correounivalle.edu.co (M.E.V.Z.)

[4] Grupo de Investigación de Fotoquímica y Fotobiología, Universidad del Atlántico, Carrera 30 Número 8-49, Puerto Colombia 081008, Colombia

* Correspondence: carlosgrande@mail.uniatlantico.edu.co; Tel.: +57-5-3599484

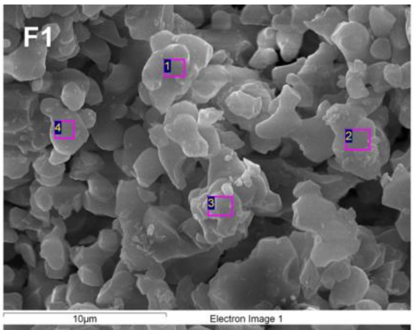

Spectrum	In stats.	C	O	P	Ca	Total
Spectrum 1	Yes	7.08	45.20	18.59	29.14	100.00
Spectrum 2	Yes		52.40	20.01	27.59	100.00
Spectrum 3	Yes	7.48	52.40	15.11	25.01	100.00
Spectrum 4	Yes		56.28	16.95	26.77	100.00
Max.		7.48	56.28	20.01	29.14	
Min.		7.08	45.20	15.11	25.01	

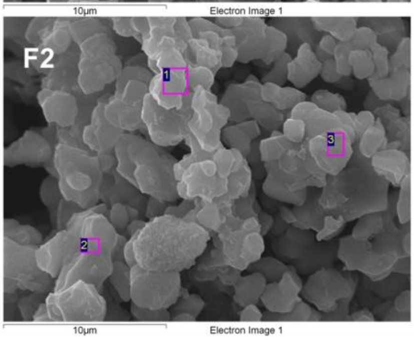

Spectrum	In stats.	C	O	P	Cl	Ca	Total
Spectrum 1	Yes		55.15	15.75	1.54	27.57	100.00
Spectrum 2	Yes		60.47	16.06		23.47	100.00
Spectrum 3	Yes	6.94	44.24	18.21		30.61	100.00
Max.		6.94	60.47	18.21	1.54	30.61	
Min.		6.94	44.24	15.75	1.54	23.47	

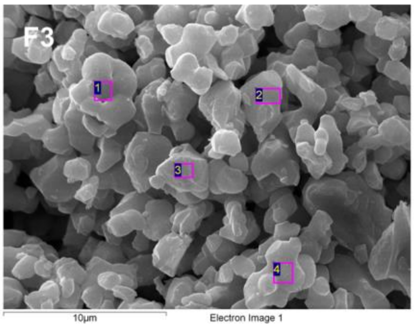

Spectrum	In stats.	C	O	P	Ca	Total
Spectrum 1	Yes	6.84	43.94	17.82	31.40	100.00
Spectrum 2	Yes	7.59	44.16	17.64	30.61	100.00
Spectrum 3	Yes		54.57	20.44	24.99	100.00
Spectrum 4	Yes		53.24	17.94	28.82	100.00
Max.		7.59	54.57	20.44	31.40	
Min.		6.84	43.94	17.64	24.99	

Figure S1. EDS for the composite CS/HA/β-TCP. F1, 87.7%β-TCP/3.5%CS/8.8%Commercial HA/0.8mL $CaCl_2$; F2, 87.7%β-TCP/3.5%CS/8.8%Commercial HA/1mL $CaCl_2$; F3, 87.7% β-TCP/3.5%CS/8.8%natural HA /0.8mL $CaCl_2$.
